# Frequency-dependent dynamics of steady-state visual evoked potentials under sustained flicker stimulation

**DOI:** 10.1038/s41598-024-59770-5

**Published:** 2024-04-23

**Authors:** Maciej Łabęcki, Maria Małgorzata Nowicka, Andrzej Wróbel, Piotr Suffczynski

**Affiliations:** 1 Department of Biomedical Physics, Faculty of Physics, University of Warsaw, 5 Pasteur St, 02-093 Warsaw, Poland; 2 Laboratory of Language Neurobiology, Nencki Institute of Experimental Biology, 3 Pasteur St, 02-093 Warsaw, Poland; 3 Laboratory of Neuroinformatics, Nencki Institute of Experimental Biology, 3 Pasteur St, 02-093 Warsaw, Poland; 4 Department of Epistemology, Faculty of Philosophy, University of Warsaw, 3 Krakowskie Przedmiescie St, 00-047 Warsaw, Poland

**Keywords:** Sensory processing, Visual system

## Abstract

Steady-state visual evoked potentials (SSVEP) are electroencephalographic signals elicited when the brain is exposed to a visual stimulus with a steady frequency. We analyzed the temporal dynamics of SSVEP during sustained flicker stimulation at 5, 10, 15, 20 and 40 Hz. We found that the amplitudes of the responses were not stable over time. For a 5 Hz stimulus, the responses progressively increased, while, for higher flicker frequencies, the amplitude increased during the first few seconds and often showed a continuous decline afterward. We hypothesize that these two distinct sets of frequency-dependent SSVEP signal properties reflect the contribution of parvocellular and magnocellular visual pathways generating sustained and transient responses, respectively. These results may have important applications for SSVEP signals used in research and brain-computer interface technology and may contribute to a better understanding of the frequency-dependent temporal mechanisms involved in the processing of prolonged periodic visual stimuli.

## Introduction

The recording and analysis of human brain responses elicited by periodic sensory stimulation dates back almost to the discovery of human electroencephalography (EEG). Soon after Berger’s publication of the first EEG in humans^1^, Adrian and Matthews recorded their own EEG responses while viewing a 30 W car headlight bulb covered with a spinning disk attached to a gramophone motor^2^. In this way, they recorded brain responses to periodic visual stimulation and observed for the first time that their rate was the same as that of the stimulus. Using an analogy to forced oscillations in physics, the recorded EEG signals were later named steady-state visual evoked potentials (SSVEP) based on the assumption of their stability in phase and amplitude over time^3^. Since their discovery, many properties and applications of SSVEP have been recognized^4,5^. E.g., it has been suggested that the relationship between SSVEP amplitude and stimulation frequency exhibits three different maxima, namely a low-frequency maximum near 10 Hz, a medium-frequency maximum around 15 Hz, and a high-frequency maximum with a peak at 25 Hz^6^. Similar but slightly different maxima at 10, 15 and 40 Hz were later identified by Regan^7^. It was shown that the medium-frequency responses (16 Hz) were sensitive to the color of the light stimuli, while the responses around high-frequency maximum (40 Hz) were mainly sensitive to light intensity; this suggested that medium- and high-frequency SSVEP were mediated by parvocellular (P) and magnocellular (M) visual pathways, respectively^8^. Indeed, selective lesions of the M and P pathways in monkeys showed that lesioning the M pathway resulted in a loss of sensitivity to high temporal frequencies, while loss of the P pathway mainly affected sensitivity to low frequencies^9^. These studies suggested that SSVEP evoked with achromatic flicker involved both visual pathways. The processing of lower frequencies is dominated by the P pathway, while stimulation with higher frequencies mainly activates the M pathway. However, it is unclear how these two visual subsystems affect the temporal properties of SSVEPs, e.g., the time course of the response.

The SSVEPs are typically examined in the frequency domain^10^ due to the periodicity of steady-state potentials. However, such an analysis is not suitable for investigating the temporal dynamics of the steady-state responses and examining their ‘steadiness.’ It is well-documented that repeated presentation of single flashes may reduce the amplitude of the transient component of evoked responses in EEG/MEG signals^11^. The time-domain changes in SSVEP amplitude or power are less recognized as the effect of prolonged periodic stimulation on EEG amplitude (or instantaneous power, being proportional to the square of amplitude samples) was investigated in only a few studies. In these studies, three different behaviors of steady-state signals evoked by a sustained stimulus were distinguished: facilitation—the increase of the SSVEP power over time, habituation—a decrement in power over time, and a stable course of the power during steady-state visual stimulation. We should note that, in the literature, habituation and adaptation are often used interchangeably. In this study, we use the term adaptation if it was used in the referenced work. Sustained stimulation by checkerboard reversal stimuli with a spatial frequency of 0.77 c/deg (check size 1.3 deg) and reversal rate 8.3 rev/s led, on average, to a fast increase followed by an exponential decline in SSVEP amplitude^12^. Another study found that the SSVEP amplitude time course depended on spatial frequency^13^. Continuous stimulation with sinusoidal grating reversing with a temporal frequency of 6.65 Hz evoked steady-state potentials that were stable for spatial frequencies of 0.77 and 1.55 c/deg, increased within 6–12 s towards a plateau for 3.1 c/deg and exhibited a fast rise and slower decrease for 6.2 and 12.4 c/deg. The authors also noted that contrast-adapted responses had shown only moderate changes in signal amplitudes compared to non-adapted SSVEP signals. SSVEP habituation has also been studied using polychromatic circular light stimulus (5 deg size) flickering at 13, 32 and 40 Hz^14^. Most of the prominent effects were obtained with continuous 50 s stimulation with green light. The response to 40 Hz flicker showed an increase in amplitude after 20 s, while those at 13 and 32 Hz exhibited habituation, particularly after 40 s. Achromatic stimulation showed only a weak habituation effect at the 13 Hz stimulation frequency. However, some significant effects in long-term amplitude modulation might have been hindered by noise, as only six subjects and a single trial of continuous stimulation were used. In the studies mentioned above, simple visual stimuli, either flicker or reversing checkerboard and grating patterns, were presented. More complex stimuli, i.e., pictures of faces with luminosity changing sinusoidally with a 3.5 Hz frequency have also been used^15^. They showed that SSVEP amplitude adapted down to 30% of the original amplitude for identical faces but was stronger and non-adapting when different faces were shown in the sequence. In both conditions, the size of each face picture varied during presentation (random size between 82 and 118% of base face size); hence, adaptation to identical faces but a lack of adaptation to different faces cannot be simply explained by adaptation to simple features, e.g., the brightness of the stimulus, rather, higher-level effects related to complex pattern recognition must have been involved in modulating the response. Among studies investigating long-term changes in SSVEP signals, only one study^14^ used different stimulus frequencies as their objective was a comparison of alpha band stimulation to less annoying gamma band stimuli applied for diagnostic purposes. In other studies, a single temporal frequency of the stimulus was used. Hence, in general, the dependence of long-term SSVEP dynamics on stimulus frequency has not been widely described.

A better understanding of these effects may have practical implications. For example, in psychology and cognitive science, SSVEP is often used to study various processes such as working memory^16^, perception^17^, attention^18^ and consciousness^19^. In brain-computer interfaces (BCI), one of the major paradigms of BCI control is based on SSVEP signals elicited by flickering stimuli delivered using a visual stimulator^20^, as well as an augmented reality setup^21^. In such applications, the user’s fatigue is an important limiting factor^22^, and it is recognized that it may depend on stimulation frequency, especially in the 5–25 Hz range^23^. In migraine patients, the temporal behavior of SSVEP amplitude often shows attenuated habituation compared to control subjects^24–26^, showing the importance of the accurate characterization of SSVEP evolution in both groups. Furthermore, 4–8 weeks of 1 h daily stimulation with combined auditory and visual gamma flicker was recently tested as a new therapeutic approach to Alzheimer’s disease^27^. Accordingly, the goal of our study was to better characterize SSVEP amplitude changes during prolonged stimulation with different frequencies. We analyzed SSVEP responses during continuous 1 min visual flicker stimulation at 5, 10, 15, 20 and 40 Hz. We used 50 stimulation runs for each frequency to obtain reliable average SSVEP power estimates. We found that responses to 5 Hz stimulation were facilitated throughout the whole stimulation period. Conversely, for higher stimulation frequency, facilitation was observed only initially, mostly within the first 5 s of stimulation. Afterward, the response amplitudes often declined considerably, especially for stimulation frequencies of 10 and 15 Hz. These changes suggest that the visual processing of rhythmic stimulation may involve a frequency-dependent mechanism or multiple interacting mechanisms acting within various time scales. Our results may contribute to the advancement of current applications of SSVEPs evoked by prolonged flicker stimulation and may help to elucidate their underlying neural mechanisms.

## Results

Significant temporal changes of the responses were obtained for all subjects with visual stimulation at 5, 10, 15 and 20 Hz. Stimulation at 40 Hz resulted in only five out of ten subjects showing significant SSVEP power changes with respect to baseline. In the remaining five subjects, we were not able to assess the SSVEP power changes during visual stimulation at 40 Hz. In general, for different subjects and frequencies, we observed some variability of averaged SSVEP responses both in terms of instantaneous power and time courses. However, we also observed that most subjects exhibited some common relationships between response amplitudes and stimulation frequencies.

**Figure 1:**
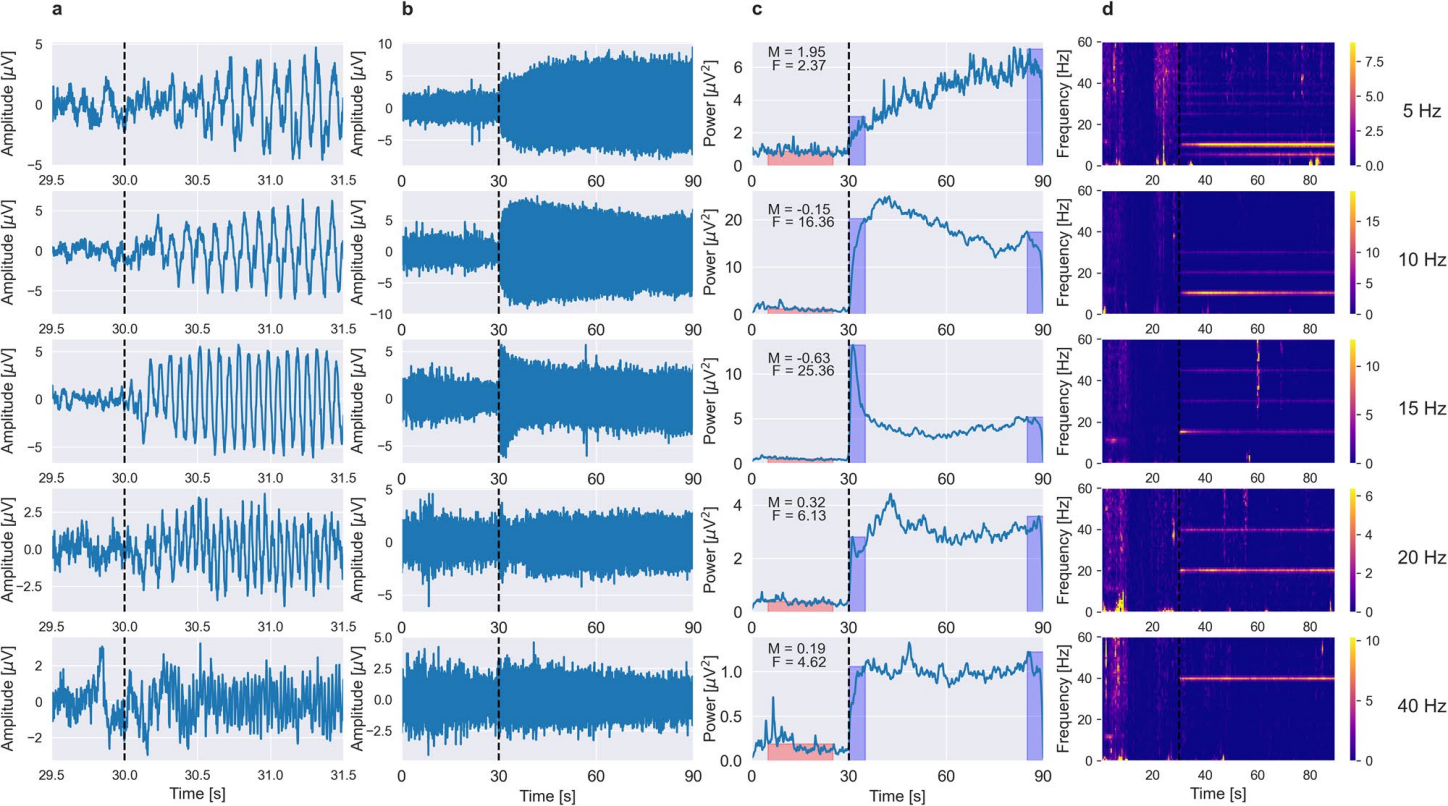
The evolution of SSVEP signals for five visual stimulation frequencies in an exemplary subject. (**a**) The raw EEG around stimulus onset (dashed black vertical line), averaged across trials shows the transient onset response for the first 250–300 ms and build-up of rhythmic response afterward. (**b**) The raw EEG averaged across trials. On each plot, the first 30 s correspond to the rest period, and the next 60 s correspond to stimulation time. The dashed black vertical line indicates stimulation onset. (**c**) The instantaneous SSVEP power averaged over 50 trials. On each panel, values of facilitation, *F* and modulation,* M* indices (see section “Methods”) are provided in the upper left corner. Red and blue rectangles show evaluation periods (width) and estimated power values (height) in the baseline (*P*_baseline_) and stimulation conditions (*P*_initial_ and *P*_final_), respectively, used for *F* and *M* calculation. It can be seen that different stimulation frequencies lead to distinct temporal evolution patterns of the power of SSVEP signals. (**d**) The spectrograms showing relative power differences from baseline (10–20 s) in the SSVEP signals. The colorbar placed along the side of each subplot indicates the mapping of the relative spectral power values (in a.u.) to color. The fundamental and higher harmonic frequencies of SSVEP responses are apparent.

Typical SSVEP responses for different stimulation frequencies are shown in Fig. 1 for a representative subject. The raw EEG around stimulus onset, averaged across trials, is shown in Fig. 1a. The transient onset response lasts for about 250–300 ms and is followed by a gradual build-up of rhythmic SSVEP signal. The raw EEG responses, averaged across trials, are shown in Fig. 1b. The long-term amplitude modulation of SSVEP for different stimulation frequencies is apparent. The instantaneous power changes averaged over 50 trials are shown in Fig. 1c. It can be noticed that the amplitude modulation shown in Fig. 1b and power changes shown in Fig. 1c exhibit some differences, e.g., for 5 Hz and 40 Hz stimulation. This is because the average SSVEP power shown in Fig. 1c was calculated by averaging the power of the bandpass-filtered SSVEP signal with energy only in a passband (see section “Methods”), while the amplitude of the average raw SSVEP signal shown in Fig. 1b was additionally influenced by the presence of harmonic frequencies and phase consistency across trials. The spectrograms showing relative SSVEP power differences from baseline (10–20 s), averaged across trials are shown in Fig. 1d. The frequency-dependent temporal changes in power of the SSVEP responses are well visible in Fig. 1c,d. For the stimulation frequency of 5 Hz, the SSVEP power gradually increased. In contrast, for both the 10 Hz and 15 Hz stimulations, power initially increased, followed by long-term habituation, with the 15 Hz stimulus demonstrating the strongest decline. The power of the responses at 20 and 40 Hz also increased initially but was mostly stable afterward.

**Figure 2:**
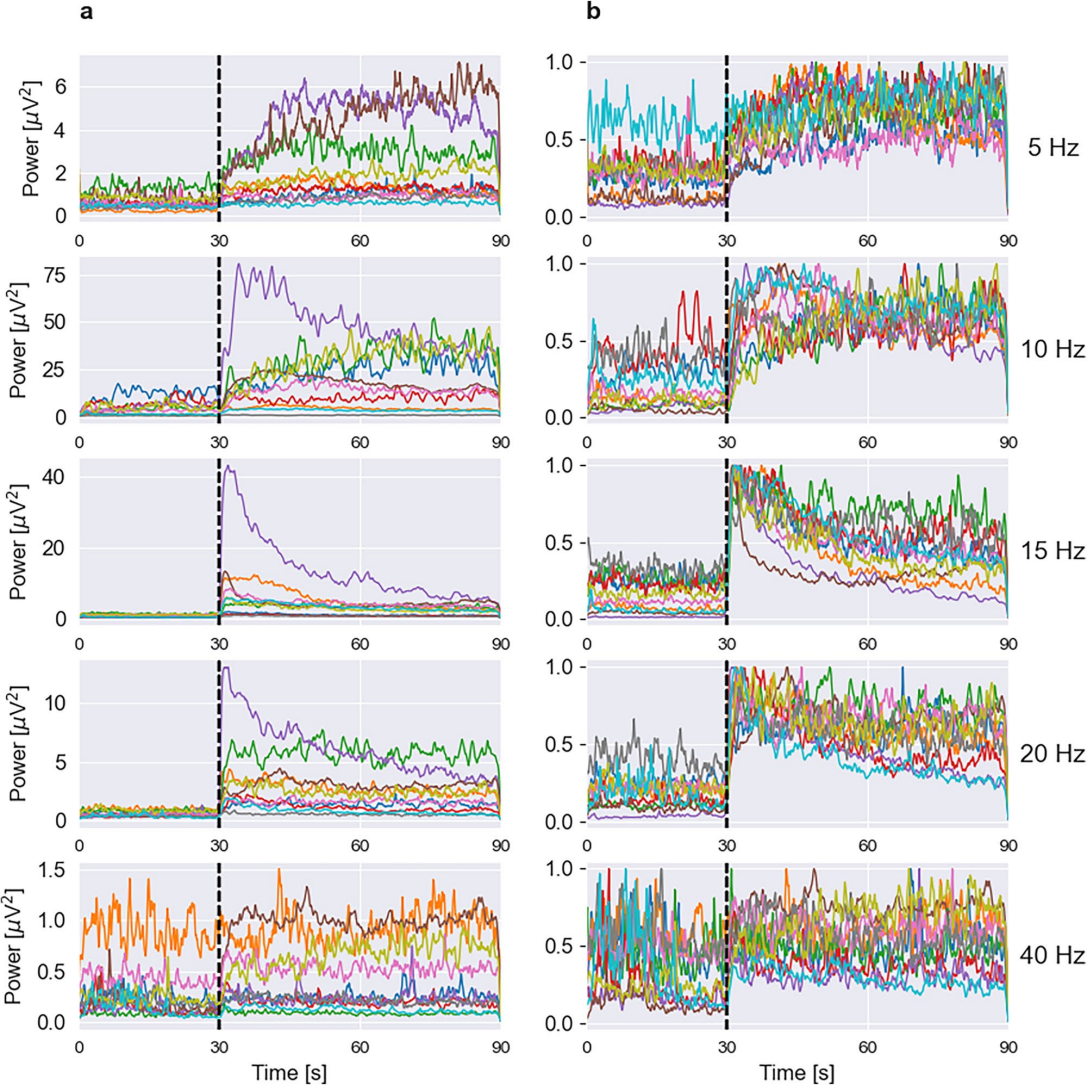
SSVEP power evolution in individual subjects. (**a**) Absolute power scale. (**b**) Normalized power scale. On each plot, the first 30 s correspond to the rest period, and the next 60 s correspond to stimulation time. The black vertical line indicates stimulation onset. Each panel corresponds to a stimulus frequency shown on the right. Each color represents SSVEP power changes in an individual subject. Despite differences in absolute SSVEP magnitude, the temporal trends in responses are specific for each frequency.

To show the consistency of our results across subjects, we show power evolution in individual subjects in a single plot for each frequency using the absolute (Fig. 2a) and normalized (Fig. 2b) scale. It can be seen that despite large differences in the absolute power of the response (Fig. 2a), the evolution trends are specific for each frequency and preserved across subjects (Fig. 2b). Responses to 5 Hz stimulus tend to steadily increase throughout the stimulation period. Responses to 15–40 stimuli exhibit a fast rise and a decline or stable behavior afterward. Only responses to 10 Hz are heterogenous exhibiting both steady rise and long-term habituation, depending on the subject. As already mentioned, in five subjects we didn’t observe significant SSVEP responses to 40 Hz stimulation. These responses are shown in Fig. 2 but are not included in the statistical analyses.

**Figure 3:**
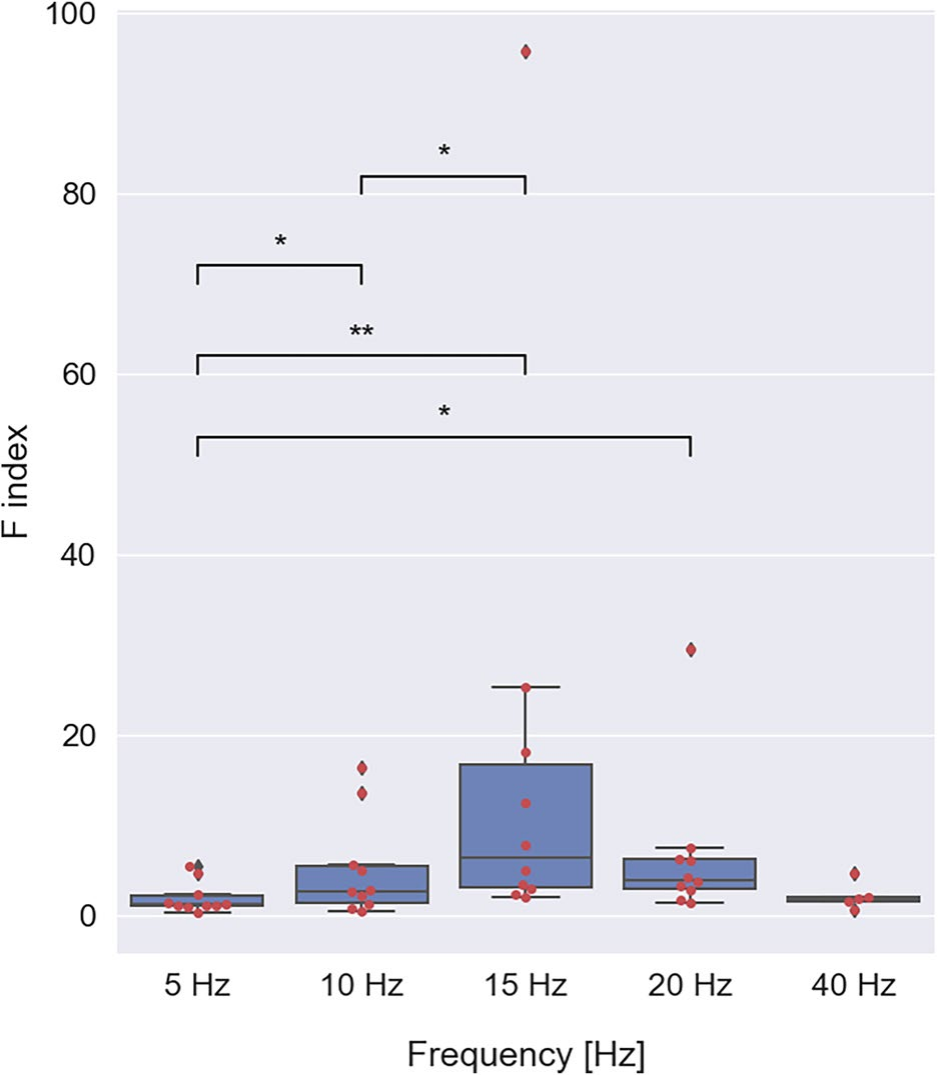
Values of the *F* index (i.e., the relative power increase during the first 5 s of stimulation) for different stimulation frequencies. Each red dot represents the *F* value of a single subject, at a given stimulus frequency. Each blue box extends from the first (*q1*) to the third (*q3*) quartile values of the data with a line at the median. The interquartile interval *iqr* = *q3* − *q1* contains 50% of the data. Whiskers of each boxplot indicate the highest and the lowest data value that is still within the lower *q1* − 1.5 * *iqr* and upper *q3* + 1.5 * *iqr* bound. Data found outside these boundaries are considered to be outliers and are shown as individual points. Significant differences were assessed by the Wilcoxon signed-rank test and are indicated by stars (*p < 0.05 and **p < 0.01).

As indicated in Figs. 1, 2 the SSVEP power typically starts to increase immediately after stimulus onset. To evaluate the initial rise in SSVEP amplitude we calculated the maximal value of the SSVEP power within the first five seconds of the stimulation period (*P*_*initial*_) and the unstimulated signal power during baseline (*P*_*baseline*_). The facilitation index *F* was defined as the relative power increase during the first five seconds of the stimulation with respect to the baseline (see section “Methods”). The overlaid swarm and boxplots of the *F* index value are shown in Fig. 3. The median *F* value reaches a maximum with the 15 Hz stimulation frequency and decreases for both lower and higher stimulus frequencies. To test whether differences in *F* values between frequencies were significant, we first tested the normality of the *F* value distributions using the Shapiro–Wilk test. For all frequencies except for 40 Hz, the *H*_*0*_ was rejected. Non-parametric Wilcoxon signed-rank test revealed statistically significant differences in *F* values for 5 versus 10 Hz (*Z* = 8.00, *p* = 0.049), 5 versus 15 Hz (*Z* = 0.00, *p* = 0.002), 5 versus 20 Hz (*Z* = 4.00, *p* = 0.014) and 10 versus 15 Hz (*Z* = 3.00, *p* = 0.010).

**Figure 4:**
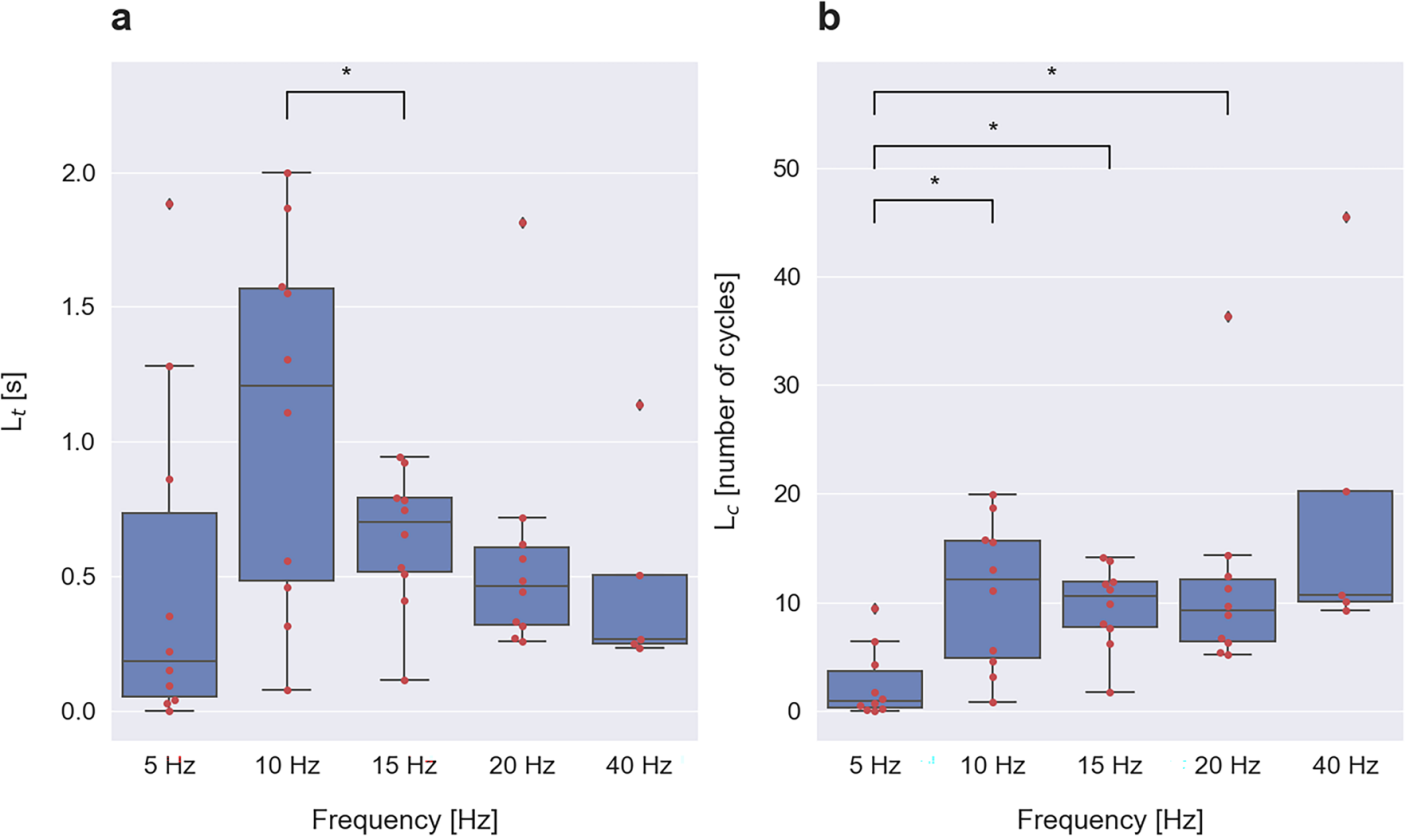
The duration of fast (phasic) SSVEP facilitation quantified by the latency of the first maximum of the SSVEP signal power derivative. (**a**) The latency expressed in seconds, *L*_*t*_. (**b**) The latency expressed as the number of cycles, *L*_*c*_. Each red dot represents the latency value of a single subject, at a given stimulus frequency. In the boxplots, each blue box extends from the first (*q1*) to the third (*q3*) quartile values of the data with a line at the median. The interquartile interval *iqr* = *q3* − *q1* contains 50% of the data. Whiskers of each boxplot indicate the highest and the lowest data value that is still within the lower *q1* − 1.5 * *iqr* and upper *q3* + 1.5 * *iqr* bound. Data found outside these boundaries are considered to be outliers and are shown as individual points. Statistically significant differences were tested by the Wilcoxon signed-rank test and paired *t*-test and are indicated by stars (*p < 0.05).

As can be seen in the exemplary subject data shown in Fig. 1c, initial facilitation induced by 10 and 40 Hz stimulation consisted of two distinct phases—a fast phase occurring typically within 1–2 s and a slower phase occurring afterward. The same phenomenon was also observed in other subjects for other stimulation frequencies but was not easily detectable visually. To distinguish the time scale of fast and slow facilitation, we computed the derivative of SSVEP signal power. Typically, within the first five seconds, the derivative increased towards a maximum and subsequently decreased. We associated the rising phase of the derivative with the fast facilitation and the falling phase of the derivative with the slower facilitation process. Accordingly, the duration of the fast facilitation phase was quantified by the *L*_*t*_ index, defined as the latency of the first maximum of the SSVEP signal power derivative (see Supplementary Fig. S1 online). The overlaid swarm and boxplots of *L*_*t*_ values for different stimulation frequencies are shown in Fig. 4a. The *L*_*t*_ index is lowest for the 5 Hz stimulation frequency, attains maximal value for the 10 Hz stimulus and decreases monotonically for higher stimulation frequencies. In the statistical analysis, the normality of the *L*_*t*_ value distributions was rejected by the Shapiro–Wilk test for 5, 20 and 40 Hz, and the Wilcoxon signed-rank test did not reveal statistically significant differences in *L*_*t*_ values among these frequencies. The only significant difference was 10 versus 15 Hz (*t*(9) = 2.45, *p* = 0.037), as determined by a paired *t*-test. The synaptic facilitation process often depends on the number of repeated stimuli^28^. Accordingly, we also looked at the duration of fast facilitation expressed as the number of stimulus cycles, *L*_*c*_. It was calculated as the *L*_*t*_ index in seconds multiplied by stimulation frequency in Hz. For 5 Hz stimulation, the fast facilitation phase lasted only one cycle. However, for higher stimulus frequencies, the fast facilitation phase was approximately constant and lasted about ten cycles (Fig. 4b). The normality of the *L*_*c*_ value distributions was again rejected by the Shapiro–Wilk test for 5, 20 and 40 Hz, and the Wilcoxon signed-rank test revealed that the *L*_*c*_ index was significantly different for 5 versus 10 Hz (*Z* = 5.00, *p* = 0.020), 5 versus 15 Hz (*Z* = 3.00, *p* = 0.010), 5 versus 20 Hz (*Z* = 3.00, *p* = 0.010) comparisons, but no significant differences were found among frequencies in the 10–40 Hz range.

**Figure 5:**
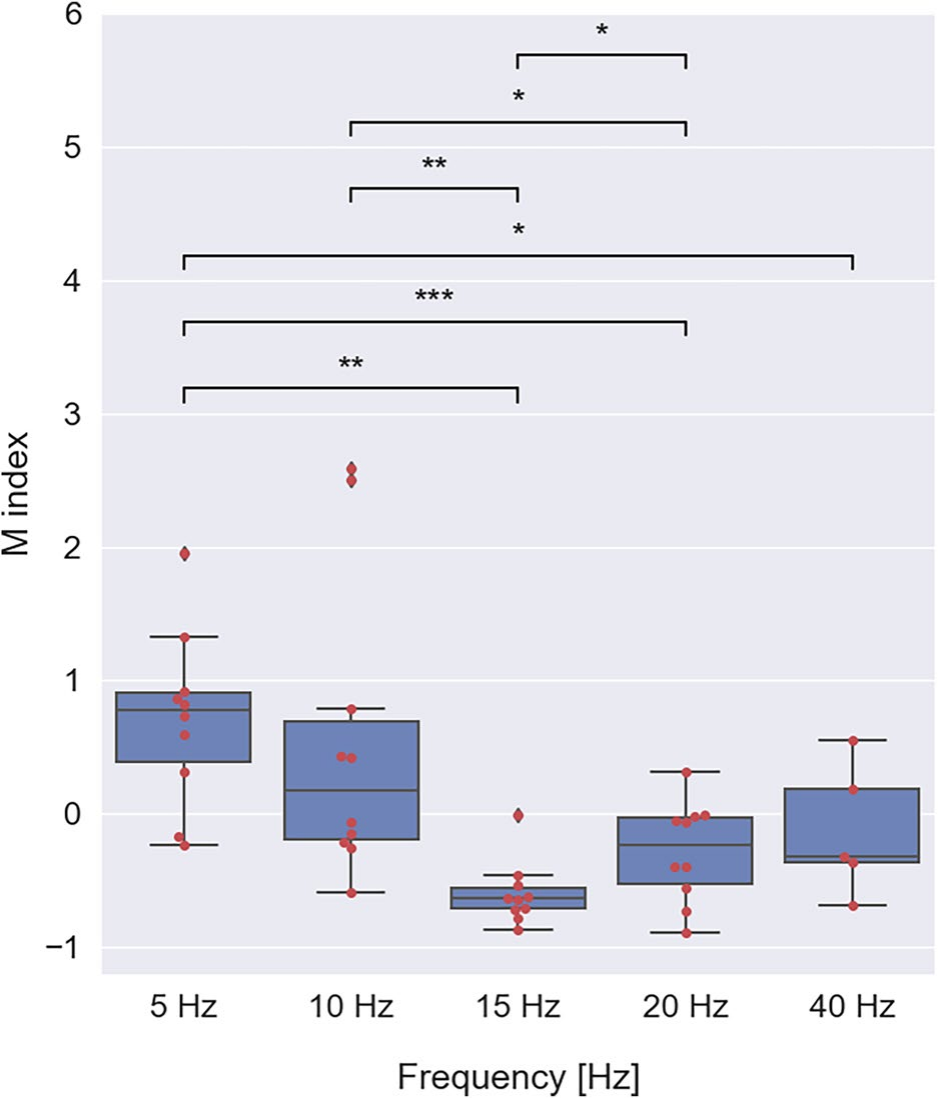
The long-term power evolution of SSVEP quantified by the *M* index. Each red dot represents the modulation index *M* of a single subject, at a given stimulus frequency. In the boxplots, each blue box extends from the first (*q1*) to the third (*q3*) quartile values of the data with a line at the median. The interquartile range *iqr* = *q3* − *q1* contains 50% of the data. Whiskers of each boxplot indicate the highest and the lowest data value that is still within the lower *q1* − 1.5 * *iqr* and upper *q3* + 1.5 * *iqr* bound. Data found outside of these boundaries are considered to be outliers and are shown as individual points. Significant differences were assessed by the Wilcoxon signed-rank test and a paired *t*-test and are indicated by stars (*p < 0.05, **p < 0.01 and ***p < 0.001). The differences in *M* values across frequencies show that the long-term properties of SSVEP signals are frequency-dependent.

To evaluate long-term SSVEP power changes we calculated the maximal value of the SSVEP power within the first and last 5 s of the stimulation period (*P*_*initial,*_* P*_*final*_) and the unstimulated signal power during baseline (*P*_*baseline*_). The modulation index *M* was defined as the relative power change during stimulation with respect to the baseline (see section “Methods”). Negative *M* index values correspond to a decrease in SSVEP power, while positive *M* values indicate an increase in SSVEP power across a whole stimulation period. The overlaid swarm and boxplots of *M* values for all stimulation frequencies are shown in Fig. 5. For the 5 Hz stimulation, the majority of subjects showed a positive *M* value, reflecting an increase in SSVEP power over time. In contrast, for the 10 Hz stimulus, the *M* values were spread across negative and positive values, meaning that all three types of long-term SSVEP power changes, described here as habituation, facilitation and stability, were possible. On the other hand, almost all responses to the 15 Hz flicker decayed during prolonged stimulation, with 9 out of 10 subjects exhibiting habituation reflected in negative *M* values. The *M* values for 20 and 40 Hz stimulations were predominantly negative, but the data points were shifted towards neutral values suggesting that in some subjects, long-term SSVEP responses were stable or in a dynamic equilibrium between initial facilitation and subsequent habituation. We also tested whether differences in power modulation described by the *M* index for different stimulus frequencies were significant. The Shapiro–Wilk normality test rejected the *H*_*0*_ hypothesis for 10 and 15 Hz frequencies, suggesting a deviation of normality of the *M* value distribution at these frequencies. Accordingly, significant differences in *M* values among 5, 20 and 40 Hz frequencies were assessed by a paired *t*-test, while tests involving 10 and 15 Hz frequencies were carried out using the Wilcoxon signed-rank test. Both tests revealed several significant differences, namely: 5 versus 15 Hz (*Z* = 55.0, *p* = 0.002), 5 versus 20 Hz (*t*(9) = 4.80, *p* < 0.001), 5 versus 40 Hz (*t*(4) = 3.12, *p* = 0.036), 10 versus 15 Hz (*Z* = 55.0, *p* = 0.002), 10 versus 20 Hz (*Z* = 51.0, *p* = 0.014), 15 versus 20 Hz (*Z* = 8.00, *p* = 0.049).

## Discussion

In this study, we addressed the question of whether visual EEG responses to periodic flash stimulation are stable or deviate from a steady-state over time. In our earlier study, we found that prolonged 15 Hz stimulation led to pronounced SSVEP long-term habituation in a majority of subjects^29^. To analyze the role of stimulus frequency in this phenomenon, in the current study, we applied flicker stimulation at five different frequencies: 5, 10, 15, 20 and 40 Hz. We found that SSVEP behavior induced by 5 Hz and by 10–40 Hz stimulation was different. Responses to the 5 Hz stimulus were characterized by a small initial facilitation, reflected by small F index values (Fig. 3), absence of fast phasic facilitation (Fig. 4) and continuous increase of power over the whole stimulation period, as evidenced by a positive *M* value in the majority of subjects (Fig. 5). In contrast, responses to higher stimulus frequencies, 15–40 Hz, showed stronger initial facilitation within the first 5 s of the stimulus (Fig. 3), exhibited fast phasic facilitation lasting 10 flicker flashes (Fig. 4b) and showed a long-term power decrease, i.e., habituation, in the majority of subjects (Fig. 5). Responses to 10 Hz stimulation were in many respects similar to responses induced by higher stimulus frequencies, i.e., 15–40 Hz. Namely, they exhibited considerable initial facilitation (Fig. 3) with fast phasic facilitation lasting a similar number of cycles (Fig. 4b). However, in terms of long-term behavior, they showed both facilitation and habituation as evidenced by the wide distribution of both positive and negative *M* values across subjects (Fig. 5). This finding may suggest that responses to the 10 Hz stimulus correspond to a ‘border case’ between evident long-term facilitation at 5 Hz and prominent long-lasting habituation at 15 Hz.

Some of our findings can be compared with the results of previous studies investigating SSVEP properties, although there is little prior research studying EEG responses to sustained flicker stimulation. In our study, the magnitude of initial facilitation, i.e., relative power increase within 5 s of stimulation, was quantified by the *F* index. The relationship between *F* and stimulation frequency exhibited a clear maximum at 15 Hz (Fig. 3). These results agree with previous findings, which analyzed the effects of short, i.e., 500 ms duration, flicker stimulation in the 5–60 Hz range on EEG responses. They showed that the amplitude of SSVEP response peaked at 15 Hz^30^, as in our study. The long-term dynamics of SSVEPs were investigated in a single-subject experiment using 10 Hz flicker stimulation presented for 40 s. The SSVEP responses showed initial facilitation during the first 12 s, subsequent habituation for another 12 s and relatively constant amplitude afterward. Another study investigated SSVEP dynamics during sustained 60 s stimulation by patterned fields with a reversal rate of 8.3 rev/s^12^. They showed that the long-term evolution of responses at 8.3 Hz (i.e., the second harmonic of the stimulus frequency) was heterogeneous among 19 subjects and showed adaptation (termed habituation in our study), lack of adaption, and a continuous increase in amplitude. These results corroborate our results for 10 Hz stimulation, showing substantial inter-individual variability at this frequency (Fig. 5). Kuo and colleagues^14^ used a single trial of 50 s long stimulation by white, red, green and blue lights flickering at 13, 32 and 40 Hz. The SSVEP responses to white light flicker habituated weakly at 13 Hz but showed no clear trend of modulation over time for 32 and 40 Hz stimulation. However, responses to color stimuli, especially green, showed a more pronounced amplitude evolution, i.e., habituation at 13 and 32 Hz and facilitation at 40 Hz. Similarly, the adaptation of the responses to a red-green grating but lack of adaptation to an achromatic grating with a spatial frequency of 0.34 c/deg and reversal rate of 7.5 Hz was reported^31^. These results do not corroborate our findings, which showed SSVEP amplitude modulation for achromatic flicker. On the other hand, another study showed that for achromatic pattern reversal stimulation with a temporal frequency of 6.65 Hz and low spatial frequencies (0.77 or 1.55 c/deg), the SSVEP amplitude was indeed constant^13^. Intermediate spatial frequencies (3.1 c/deg) led to an increase in amplitude, while high spatial frequencies (6.2 and 12.4 c/deg) led to its initial increase, followed by a marked decrease. This shows that SSVEP evolution depends on both the temporal and spatial frequency of the stimulus, and it is difficult to directly compare our findings obtained with unpatterned flashing stimulus with those obtained with patterned stimuli for different spatial frequencies.

The mechanisms responsible for the different temporal evolutions of SSVEP signals evoked by 5 Hz and 10–40 Hz flicker stimuli are unclear. The contrasting SSVEP behavior observed for low and high stimulus frequencies cannot be solely explained by a temporal decrease in attention or an increase in fatigue levels. These two processes are expected to decrease response magnitude, irrespectively on stimulus frequency. On the other hand, the subjective comfort level was lowest for visual stimulus frequencies between 10 and 30 Hz^32^, which is the frequency range where the strongest habituation occurred in our study (Fig. 5). Responses of brain activity due to repetitive stimulation (not necessarily periodic) are usually reduced, and multiple mechanisms, such as firing-rate adaptation, synaptic depression and potentiation, were proposed to be involved in this phenomenon^11^. Such changes may take place at various levels of the visual processing stream. Pattern reversal was shown to induce habituation in the retinal ganglion cell responses, possibly due to their limited energy-storage capacity and high metabolic demand during stimulation^33,34^. On the other hand, electroretinogram adaptation was observed only with pattern stimulation but not with an 8 Hz flicker stimulus^35^. The authors suggested that pattern and flicker electroretinograms might originate from different retinal neurons. Furthermore, in migraine patients, the habituation of visual-evoked potentials in response to pattern reversal stimulation is often impaired^25,36^. Still, it can be normalized by transcranial magnetic stimulation (rTMS)^37^, suggesting a thalamocortical rather than a retinal origin of the habituation phenomenon. Lindström and Wróbel^38^ showed that cortico-geniculate synapses exhibit a three–fourfold EPSP potentiation at the 10–50 Hz frequency range. This mechanism can support intrinsic corticothalamic oscillations^39^ and could be responsible for phasic facilitation of SSVEP during the first two seconds of stimulation and accompanied by parallel, long-lasting modulatory mechanisms, e.g., recurrent inhibition via perigeniculate neurons^40^.

The visual system in primates consists of parallel parvocellular (P) and magnocellular (M) pathways that mediate different visual functions^41^. Cortical neurons of both pathways respond with sustained and transient characteristics, respectively^42^. Selective lesion studies in primates showed that lesions of the M pathway resulted in the loss of contrast sensitivity at high temporal frequencies, while loss of the P pathway mainly affected contrast sensitivity at low temporal frequencies^9^. Accordingly, responses to low temporal stimulation frequencies, mediated by the non-adapting P pathway, should exhibit less attenuation than responses to higher frequencies relayed by the fast-adapting M subsystem. Indeed, strong high-contrast adaptation of M cells but not P cells was found in the lateral geniculate nucleus of macaques^43^. By simultaneously observing inputs from the ganglion cells, the authors suggested a retinal origin of M cell adaptation. Furthermore, the application of the steady- and pulsed-pedestal paradigms showed that flicker adaptation was related to desensitization of the M pathway but not the P pathway in humans^44^. Additionally, the reduced sensitivity of the M pathway was larger for 10 Hz than for 2 Hz stimulation, which is in agreement with another study^45^ and our results.

Finally, it should be noted that neural activity changes during photic stimulation were accompanied by decreases in glucose and increases in lactate levels in the visual cortex^46,47^, showing that SSVEP evolution may also depend on neurometabolic coupling. We cannot also exclude that long-term changes observed in SSVEP response amplitudes were related to changes in pupil size. Indeed, there is some evidence showing that habituation of the flash-evoked responses measured in the visual cortex may depend on pupil size and may disappear when the pupil is dilated with atropine^48,49^. In addition, it is worth noting that a 60 s flicker stimulus at 2–64 Hz frequency led to an increase in retinal vessel diameters^50^, which might lead to an increase in neural activity. The vasodilation was most pronounced at 4 Hz stimulation, which could be linked with the long-term facilitation of SSVEP at 5 Hz observed in our experiments.

Overall, we showed that EEG responses to prolonged visual stimulation in the 5–40 Hz frequency range can exhibit two distinct patterns. Responses to low, i.e., 5 Hz frequencies, showed small or absent initial phasic facilitation but continued to increase over 60 s of stimulation. Responses to higher, i.e., 10–40 Hz stimulus frequencies, exhibited marked fast phasic facilitation during the first stimulation cycles and tended to habituate during sustained stimulation. We associate these specific SSVEP properties with distinct characteristics of parvocellular and magnocellular neurons in the visual system. Our results may contribute to a better understanding of the temporal processing of sustained periodic inputs widely used in psychological studies and BCI applications.

## Methods

### Subjects and data collection

Ten healthy participants (five females) between the ages of 22 and 30 (M = 25.9; SD = 3.5) took part in the study. All participants had normal or corrected to normal vision and reported no history of mental or neurological disorders. All participants were right-handed. The study was conducted with the approval of the Rectors Committee for Ethics of Research with Human Participants at the University of Warsaw. All experiments were performed according to the ethical standards of the institutional and national research committee. Informed consent was obtained from all participants. EEG signals were recorded using a TMSi Porti 7 amplifier with 21 electrodes mounted on an EEG cap using the 10–20 system. The sampling frequency was 512 Hz. The ground electrode was placed on the AFz position, and the average earlobe signal was used as the reference signal. An output from a photodiode measuring flickering light was recorded simultaneously with the EEG. The results are presented using data averaged from O1 and O2 channels.

### Stimuli and procedure

Participants were seated in a dark room at a viewing distance of 100 cm from the custom-made SSVEP stimulator. The stimulator consisted of an arbitrary waveshape generator and a lighting panel, which was backlighted by a diode. The maximum luminance of the panel was 600 cd/m^2^. The panel size was 10 × 10 cm, corresponding to the visual angle of 5.7 deg, both vertically and horizontally. The participants were instructed to keep focused attention on the entire flickering field. The temporal modulation of the stimulus was described by a square-wave function with a 50% duty cycle. The experimental paradigm was based on our previous study^29^. A single run, also referred to as a trial, lasted 90 s and consisted of 30 s of rest and 60 s stimulation periods (see Supplementary Fig. S2 online). During the rest period, the stimulation was switched off, and the subjects were instructed to keep their eyes open. We used five different stimulation frequencies, i.e., 5 Hz, 10 Hz, 15 Hz, 20 Hz and 40 Hz. For each subject, we used 50 experimental runs for each frequency, giving 250 runs for each participant. Presenting such a number of trials in one continuous sequence would last over six hours. This could be too exhausting for the subjects and could influence our results by triggering some additional long-term effects. Therefore, we decided to present trials in ten experimental sessions presented over five days with two sessions per day. During each session, 25 trials of 90 s were presented to a participant leading to a session duration of 37 min. Each session involved five repetitions of each of the five stimulation frequencies. The order of frequencies was chosen randomly for each session and participant.

### Preprocessing

The EEG signal was filtered with a first-order Butterworth filter, a high pass filter with a 2 Hz cut-off frequency and a notch filter. The EEG data were segmented into 90 s long trials with respect to the photodiode signal. The artifacts were rejected based on visual inspection. To investigate SSVEP power changes over time, instantaneous power was calculated as follows. First, the EEG signal was band-pass filtered around the stimulation frequency *Fs*, i.e., within a band [*Fs* − 0.5 Hz, *Fs* + 0.5 Hz] using a second-order Butterworth filter. Next, the amplitude of the filtered signal was squared to obtain a band power signal around the *Fs* frequency. Afterward, the smooth envelope of the oscillating band power signal was estimated using a low-pass third-order Butterworth filter with a cut-off frequency of 5 Hz. Finally, the envelopes were averaged across all trials for a given stimulation frequency in a given subject, resulting in an average SSVEP signal power at that frequency in that subject. We should note that there are alternative ways to estimate SSVEP power changes over time, e.g., calculating the average SSVEP signal, band pass filtering of the average signal and squaring its amplitude. However, this method requires high trial-to-trial synchrony which is not guaranteed due to jitter having intrinsic (brain) or extrinsic (equipment) sources. Therefore, the power estimates based on trial-averaged SSVEPs might be inaccurate. Our method of first calculating the power of individual trials and next calculating the average power across all trials is more robust against the jitter factor and may lead to a more accurate characterization of temporal power changes in SSVEP signals.

### Quantification of the responses

The initial increase in SSVEP amplitude often consisted of fast, phasic facilitation followed by a slower rise in amplitude. The fast and slow facilitation periods are clearly visible in Fig. 1 in response to 10 and 40 Hz stimulation. The initial rise in response amplitude, including these two stages, was quantified by the facilitation index *F*, representing the relative power increase within the first 5 s of stimulation:

1 $$ F = \, \left( {P_{initial} {-}P_{baseline} } \right)/P_{baseline} $$

where *P*_*initial*_ is the maximal value of SSVEP power within the first 5 s of the stimulation period, and *P*_*baseline*_ is the SSVEP power averaged from the fifth to twenty-fifth s of a baseline signal. Additionally, to distinguish distinct facilitation phases, we analyzed the SSVEP signal power derivative, which typically sharply increased during fast phasic facilitation, reached a maximum within the first second and declined afterward. Accordingly, the characteristic duration of the fast facilitation process was assessed by the *L*_*t*_ index, defined as the latency (in seconds) of the first maximum of the SSVEP signal power derivative (see Supplementary Fig. S1 online). We also analyzed the duration of the fast facilitation phase expressed as the number of stimulation cycles, *L*_*c*_, which was calculated as the *L*_*t*_ index multiplied by stimulation frequency, *F*_*s*_. Long-term SSVEP power changes were quantified by a modulation index *M* introduced earlier^29^:

2 $$ M = \frac{{P_{final} - P_{initial} }}{{P_{initial} - P_{baseline} }}, $$

where *P*_*initial*_ and *P*_*baseline*_ are defined as above, and *P*_*final*_ is the maximal value of SSVEP power within the last 5 s of the stimulation period. According to this definition, negative *M* index values correspond to a decrease in SSVEP power, while positive *M* values indicate an increase in SSVEP power across a whole stimulation period with respect to the power attained within the first 5 s of stimulation.

### Statistical analysis

For some subjects and stimulus frequencies, no clear SSVEP response was observed. Accordingly, for each subject and stimulus frequency, the significance of the SSVEP response was tested using a bootstrap method. That is, we tested the null hypothesis that the average SSVEP power during baseline and stimulation periods were equal. If the hypothesis was rejected, we considered that SSVEP power differed significantly from signal power during baseline, and the response to stimulation was quantified as described above. On the other hand, if a certain stimulus frequency did not induce a significant SSVEP response in a certain subject, that particular SSVEP response in that subject was not quantified and was excluded from further analysis. To test differences in *F*, *L*_*t*_*, L*_*c*_ and *M* indices obtained for different stimulus frequencies, the sample’s normality was first tested using the Shapiro–Wilk test. Next, all pairwise comparisons between group levels using the two-tailed Wilcoxon signed-rank test or two-tailed paired *t*-test were carried out. The significance level ‘*p*’ was set to 0.05. As the number of tests for each index was not large, i.e., 10, we did not correct for multiple comparisons to avoid a type II error^51^.

### Supplementary Information

Supplementary Figures.

## Data Availability

Upon acceptance of the manuscript, the dataset will be freely available from the public repository https://osf.io together with contact information to one of the authors (M.L.) labecki@poczta.fm.
