# Supplementary material for: Frequency-dependent dynamics of steady-state visual evoked potentials under sustained flicker stimulation
Source: Sci Rep. 2024 Apr 23;14:9281. doi: 10.1038/s41598-024-59770-5 (PMC11039735; doi:10.1038/s41598-024-59770-5)
Supplement: Supplementary file 1 — Supplementary Figures. [file 41598_2024_59770_MOESM1_ESM.pdf]

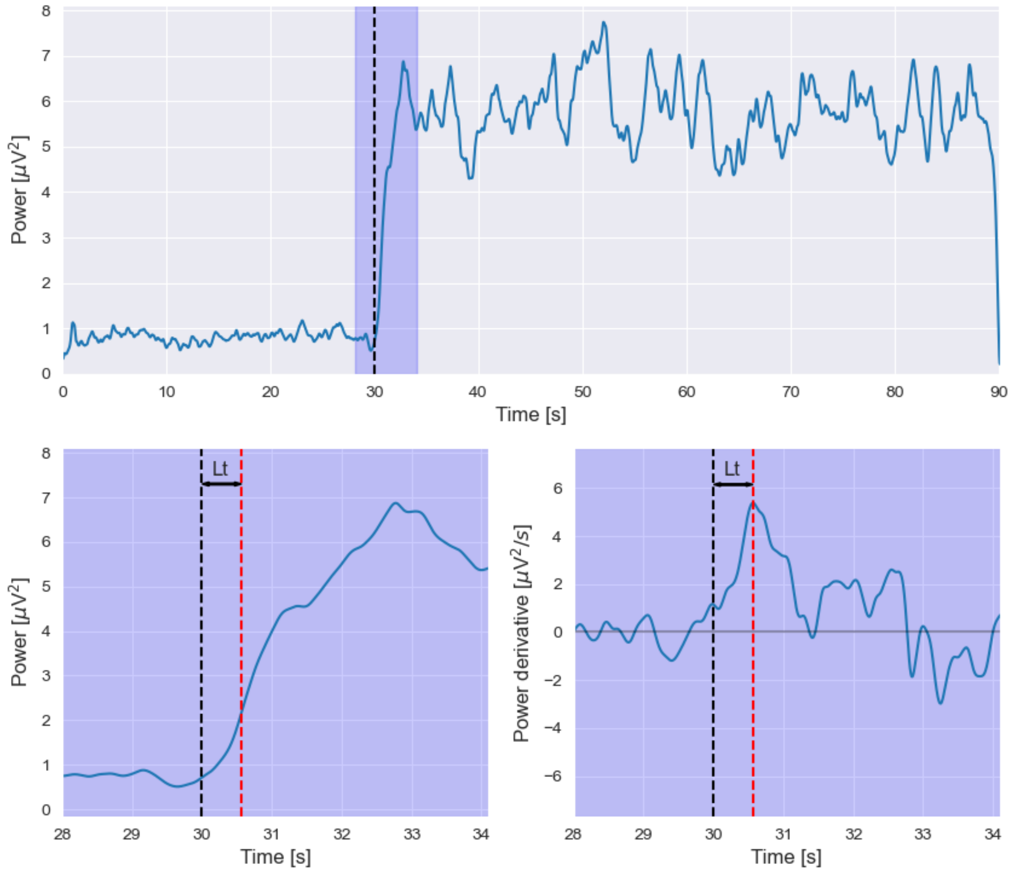

Supplementary Figure S1. Methodology of fast phasic facilitation analysis. Top panel: the instantaneous SSVEP power averaged over 50 trials with 20 Hz simulation, in a single subject. The first 30 s correspond to the rest period, and the next 60 s correspond to stimulation. The dashed black vertical line indicates stimulation onset. The blue rectangle extending from seconds 28 to 34 shows the analysis period. The bottom panel, left: 28-34 s zoom of the top panel signal. The bottom panel, right: the derivative of the signal shown in the left bottom panel. The first maximum of the power derivative is marked with the dashed red vertical line. The latency L_t is calculated as the time between the stimulation onset and the first maximum of the power derivative and is marked by an arrow between the dashed lines. The latency expressed as the number of cycles, L_c, is calculated as L_t multiplied by the stimulus frequency. For 20 Hz stimulation in this subject, the duration of fast phasic facilitation lasts around 500 ms, giving L_c equal to 10 cycles (0.5 s * 20 Hz).

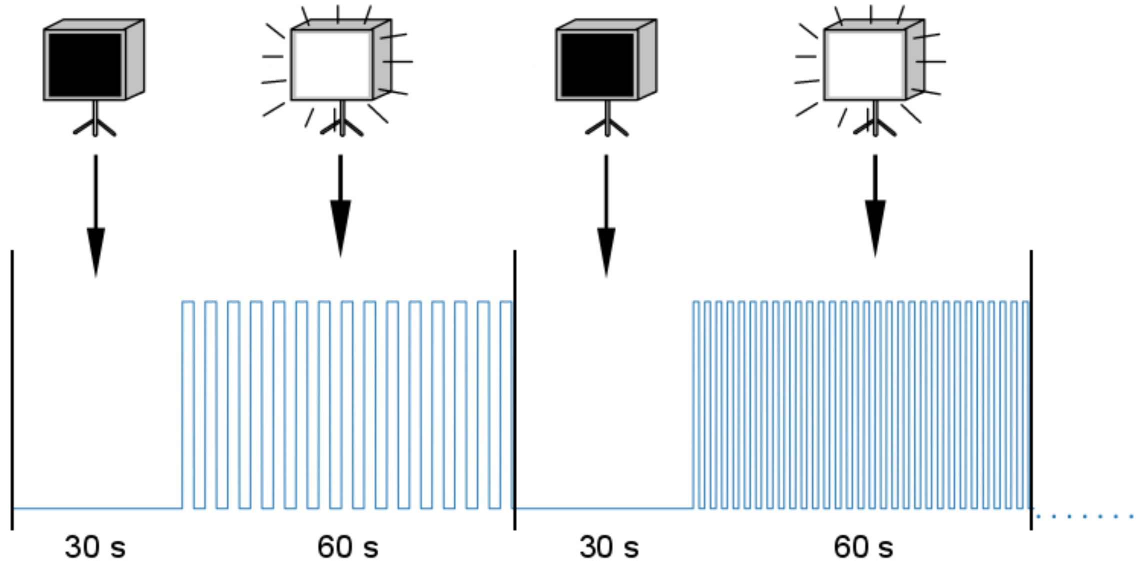

Supplementary Figure S2. The scheme of the experimental paradigm. Two exemplary trials separated by a vertical black line are presented. Each trial consists of a 30 s rest period without a light on the stimulation screen and 60 s of light flicker. The blue line schematically shows changes in screen luminosity for two different frequencies (not to scale).
